# Comparison of trigeminal neuralgia radiosurgery plans using two film detectors for the commissioning of small photon beams

**DOI:** 10.1120/jacmp.v14i6.3824

**Published:** 2013-11-04

**Authors:** Karina P. Esparza‐Moreno, Olivia A. Garcáa‐Garduño, Paola Ballesteros‐Zebadúa, José M. Lárraga‐Gutiérrez, Sergio Moreno‐Jiménez, Miguel A. Celis‐López

**Affiliations:** ^1^ Escuela de Medicina Universidad Autónoma del Estado de México Estado de México Mexico; ^2^ Laboratorio de Fásica Médica Instituto Nacional de Neurologáa y Neurocirugáa Mexico City Mexico; ^3^ Unidad de Radioneurocirugáa Instituto Nacional de Neurologáa y Neurocirugáa Mexico City Mexico

**Keywords:** trigeminal neuralgia, dose distributions, EBT2 radiochromic film, X‐OMAT radiographic film

## Abstract

Trigeminal neuralgia (TN) is a chronic, episodic facial pain syndrome that can be extremely intense, and it occurs within the regions of the face that are innervated by the three branches of the trigeminal nerve. Stereotactic radiosurgery (SRS) is the least invasive procedure to treat TN. SRS uses narrow photon beams that require high spatial resolution techniques for their measurement. The use of radiographic or radiochromic films for small‐field dosimetry is advantageous because high spatial resolution and two‐dimensional dose measurements can be performed. Because these films have different properties, it is expected that the calculated dose distributions for TN patients will behave differently, depending on the detector used for the commissioning of the small photon beams. This work is based on two sets of commissioned data: one commissioned with X‐OMAT V2 film and one commissioned with EBT2 film. The calculated dose distributions for 23 TN patients were compared between the commissioning datasets. The variables observed were the differences in the half widths of the 35 and 40 Gy isodose lines (related to the entrance distance to the brainstem) and the volume of the brainstem that received a dose of 12 Gy or more (V12). The results of this comparison showed that there were statistically significant differences between the two calculated dose distributions. The magnitudes of these differences were up to 0.33 mm and 0.38 mm for the 35 and 40 Gy isodose lines. The corresponding difference for the V12 was up to 2.1 cc. It is clear that these differences may impact the treatment of TN patients, and then it must be important to perform this type of analysis when observing complication rates. Clinical reports on irradiation techniques for trigeminal neuralgia should consider that different detectors used for commissioning treatment planning systems might result in small but significant differences in dose distributions.

PACS number: 87.55.km

## I. INTRODUCTION

Trigeminal neuralgia (TN) is a chronic, episodic facial pain syndrome that occurs in the mandibular, maxillary, and orbital regions of the face, which are innervated by the three branches of the trigeminal nerve.^(1)^ Medical therapy is the first line of treatment. However, the effectiveness of medication decreases over time, and it can produce adverse side effects.^(2)^ Surgical treatments, such as microvascular decompression, radiofrequency rhizotomy, glycerol rhizotomy, balloon compression, and nerve sectioning, have inherent surgical risks including bleeding, cerebrospinal fluid leakage, and infection.^(3)^ Stereotactic radiosurgery (SRS) is the least invasive procedure and has been demonstrated to produce significant pain relief with minimal side effects.^(^ ^2^ ^,^ ^4^, ^5^, ^6^, ^7^, ^8^, ^9^, ^10^ ^)^ SRS is an alternative treatment for patients who have failed previous neurosurgical therapy and for patients who may not tolerate surgical intervention.^(3)^ Gamma Knife (Elekta, Stockholm, Sweden) has been the standard radiosurgery technique for trigeminal neuralgia treatments. This technique uses 201 ${}^{60}\text{Co}$ sources to irradiate the trigeminal nerve. In contrast, linear accelerator (linac)‐based radiosurgery uses multiple arcs to deliver the dose to the isocenter.^(11)^ There are three main trigeminal nerve targets that have been or are being used in stereotactic radiosurgery for the treatment of TN: the gasserian ganglion, the cisternal nerve just proximal to the gasserian ganglion, and the dorsal nerve entry zone adjacent to the pons.^(2)^ Because the target can be as small as 2 mm in diameter and the required radiation dose is on the order of 80 Gy,^(12)^ narrow photon beams are used. Commonly, the evaluation of the treatment plan of a SRS for TN is performed using the isodose lines (IDLs) that encompass the target and organs at risk. Details of the criteria for the evaluation of this type of treatment plan are shown in the Methods and Materials section A below.

For dosimetry of small photon beams which have lateral electronic disequilibria and steep dose gradients in a large portion of the fields, high‐resolution measurement techniques are required.^(13)^ The greatest challenge in the dosimetry of SRS fields is the correct selection of the detector for the commissioning of the small fields.^(14)^ The use of radiographic or radiochromic films for small photon beam dosimetry is advantageous because of their high spatial resolution and the ability to perform two‐dimensional (2D) dose distribution measurements.^(15)^ Radiochromic film is particularly attractive because its effective atomic number is close to the effective atomic number of water, it is relatively insensitive to ambient light, it is self‐developing, and it can be immersed in water.^(16)^ In contrast, radiographic film is not tissue equivalent (being mostly made of silver halide), it has strong energy dependence, and it cannot be exposed to light. The response of radiographic film is a function of developing time, temperature, agitation, and developer characteristics.^(17)^ Because of these differences in the films' properties, there may be differences in the dose distributions calculated by a treatment planning system (TPS) whose commissioning data are based on this type of film, which in turn would then affect the evaluation of a TN SRS treatment plan.

However, radiographic film was widely used for the dosimetry of small photon beams approximately ten years ago.^(18)^ At present, many centers around the world may have treated TN patients with beam data commissioned with radiographic film.

The goal of this work is to assess the behavior of the isodose lines and their effect on the treatment plan evaluation of a TN radiosurgery by comparing the calculated dose distributions based on beam commissioning data acquired with radiographic and radiochromic films for a set of patients who underwent SRS for TN. The treatment plans for 23 patients were used in this study to compare the dose distributions calculated with the TPS. The beam data used for these treatments were originally commissioned with radiographic film. A new set of beam data was commissioned with radiochromic film and used by the same TPS to calculate new dose distributions for these patients. The resulting dose distributions were compared using the IDLs to evaluate the treatment plans.

## II. MATERIALS AND METHODS

### A. Trigeminal neuralgia patient dose distributions

Twenty‐three patients with TN underwent SRS with a dedicated linac (Novalis, BrainLAB, Feldkirchen, Germany) between September 2004 and September 2009. Table 1 shows the patients' demographic characteristics. The TPS used for the treatment planning of these patients was BrainSCAN v. 5.21 (BrainLAB). The beam commissioning data used for the calculation of the dose distributions of these patients were based on radiographic film X‐OMAT V2 (Kodak, Inc., Rochester, NY). These beam data were acquired in 2001. The TPS allows the use of multiple sets of commissioned data or ‘beam data profiles”. The default data profile must be set before the clinical treatment plan dose calculation. For the purposes of this work, two beam data profiles were defined for each film detector in the TPS: an ‘X‐OMAT‐beam‐profile” and an “EBT2‐beam‐profile”. All of the patients had computed tomography (CT) images (voxel size of 0.7 mm) taken using a CT scanner (Hi‐Speed, GE Healthcare, Waukesha, WI) and Siemens Somatom Sensation (Siemens AG, Munich, Germany), and several sequences (T2, T1, Fatsat) of magnetic resonance images were obtained with a Signa Excite 3T scanner (GE Healthcare). The delineated volumes of the brainstem and trigeminal nerve ranged from 2.8 to 25.96 cm^3^ and 0.05 to 0.8 cm^3^, respectively. Variations in brainstem volume were noticeable because the clinicians delineated only a small region of the brainstem that is closer to the trigeminal nerve to generate a more rigorous percent dose calculation. Noncoplanar arcs numbering between 9 and 12 were used in the treatments.

**Table 1:** TN patients' characteristics

		*No. Patients (%)*
Gender	Female	16 (69.5%)
	Male	7 (30.4%)
	75	3 (13.04%)
Dose (Gy)	85	16(69.57%)
	90	4(17.39%)
	7.5	16(69.5%)
Collimator diameter (mm)	6	6(26.1%)
	4	1(4.3%)^a^

a Not enough patients for statistical analysis.

The literature on radiosurgery for TN defines the isocenter position based on the IDL touching the brainstem surface in an attempt to limit the radiation dose delivered to the entire structure.^(19)^ Thus, the evaluation of a TN radiosurgery plan is based in the entrance distance of the IDL into the brainstem. Goss et al.^(2)^ reported that for all of the patients who were prescribed 90 Gy for the isocenter at the nerve entry zone, the 50% IDL is generally outside of the brainstem and the 30% IDL is tangential to the brainstem (i.e., the 45 and 27 Gy IDLs are outside of the brainstem and tangential to the brainstem, respectively). Maesawa et al.^(4)^ defined the isocenter position such that the 30% IDL touched the pons. Pollock et al.^(5)^ described the 20% IDL as the median IDL at the brainstem surface when delivering 90 Gy and the 40% IDL when delivering 70 Gy. At the University of California at Los Angeles (UCLA), the isocenter was placed with the 50% IDL adjacent to the region where the trigeminal nerve enters the brainstem.^(19)^ At our institution, treatment plans are prescribed to deliver 85 Gy to the isocenter, and the 47% IDL (~40Gy) is 4 mm inside the brainstem.

### B. EBT2 film dosimetry

In this study, GAFCHROMIC EBT2 films were used for all of the experimental measurements (ISP Corporation, Wayne, NJ). The commissioning of the stereotactic beams was performed according to the TPS manufacturer's specifications.^(20)^ Radiographic commissioning data were measured in 2001 using X‐OMAT V2 Kodak film following the American Association of Physicists in Medicine (AAPM) TG‐69 report.^(21)^

#### B.1 Film processing and analysis

The films were handled in accordance with the procedures outlined in the American Association of Physicists in Medicine (AAPM) TG‐55 report^(22)^ and the recommendations proposed by Butson,^(23)^ Lynch,^(24)^ and Richley.^(25)^ The sheets of film were cut into $3\times 3\;{\text{cm}}^{2},6\times 6\;{\text{cm}}^{2},\;\text{and}\;10.1\;\times \;12.7\;{\text{cm}}^{2}$ pieces 48 hr before irradiation to minimize response changes due to mechanical stress. Each piece was marked prior to cutting to preserve the orientation of the film. All of the irradiated films were digitized before exposure and 72 hr after exposure to ensure full color growth.^(26)^ Film digitization was performed using a flatbed document scanner (Epson Perfection V750; US Epson, Long Beach, CA) in transmission mode with all of the postprocessing and color management options turned off. The scanner was warmed up for 30 min before digitization and was not used for more than 2 hr continuously to avoid lamp and film heating.^(24)^ All the film pieces were positioned in the middle of the scanner bed and scanned in landscape orientation. A black template was used to ensure the same location on the scanning bed and to minimize light source scatter during scanning.^(15)^ The resolution of all of the scans was set at 300 dpi. The scanned image data were stored in 48‐bit red‐green‐blue (RGB 16 bits per color channel) and saved in the uncompressed tagged image file format (TIFF). The scans were analyzed with the red channel only due to the higher sensitivity of the film in the red region of the visible spectrum. The analysis was performed using the ImageJ^(27)^ software package. With ImageJ, a Wiener filter with a 7×7 pixel size was applied to all of the images to reduce the image noise, and a scanner background value of 613 was subtracted from the films. The film was processed according to the procedures described in the literature.^(^ ^28^ ^,^ ^29^ ^)^

#### B.2 Film calibration and beam commissioning

A 6 MV photon beam produced by the Novalis linac was used to irradiate a $30\times 30\times 30\;{\text{cm}}^{3}$ water phantom (MP3‐XS; PTW, Freiburg, Germany) with the films. In all of the measurements, $3\times 3\;{\text{cm}}^{2}$ films were used unless otherwise stated. The linac was calibrated such that 1 cGy per monitor unit was delivered at 5 cm depth with a $10\times 10\;{\text{cm}}^{2}$ field size, and the source‐to‐axis distance (SAD) was 100 cm. All of the irradiations were performed perpendicularly to the film plane. A perfectly calibrated linac was assumed (i.e., no corrections for daily variations in its output were made) (variations<0.8%onaverage). The absolute doses were measured following the International Atomic Energy Agency (IAEA) TRS 398 formalism^(30)^ in the water phantom with a 0.6 cm^3^ PTW‐30013 Farmer ionization chamber calibrated according to a traceable laboratory standard (PTW) with the same film set‐up conditions. Film calibration was performed by placing the films at a water depth of 5 cm and irradiating them with a $10\times 10\;{\text{cm}}^{2}$ field size covering the dose range 0–700 cGy. The tissue maximum ratios (TMRs), off‐axis ratios (OARs), and output factors (OFs) were measured using circular collimator projecting fields of 6.0 and 7.5 mm in diameter at the isocenter. In all of the cases, the jaws were set to a $4\times 4\;{\text{cm}}^{2}$ field size. For each collimator, the TMRs were measured at 0, 2, 4, 6, 8, 10, 12.5, 20, 45, 100, 130, 160, 190, 220, and 250 mm depths. The OFs and OARs were obtained at depths of 1.5 cm and 7.5 cm, respectively.^(20)^ The same measurements were performed with a stereotactic diode (SFD; IBA Dosimetry, Schwarzenbruck, Germany) and were performed to compare the OF, OAR, and TMR measurements to the EBT2 measurements. The calculation of the dose distribution by TPS is only affected by the OAR measurements because OFs and TMRs are used only for monitor unit (MU) calculations. The TPS used the tabulated TMRs to perform the calculations.

After EBT2 film beam commissioning, an end‐to‐end test was performed for TPS quality assurance. For that purpose, a radiosurgery head phantom (model 605; CIRS, Norfolk, VA) was used. A piece of EBT2 film $\left(6\times 6\;{\text{cm}}^{2}\right)$ was placed in the phantom. CT images were obtained with a CT scanner (Siemens Somatom Sensation; Siemens) with an image slice spacing of 0. 75 mm. The film received an additional 21±1 mGy due to CT scanning.^(31)^ A treatment plan was defined with four arcs spaced using a 6.0 mm collimator to deliver a 6 Gy radiation dose to the isocenter. The dose delivered to the radiochromic film was scaled down to avoid film overexposure. The dose distribution calculated by the TPS was exported for comparison with the dose distribution measured with the EBT2 film at the head phantom. After irradiation of the head phantom, a gamma index analysis^(32)^ was performed using DoseLab v. 4.11 (Mobius Medical Systems, Houston, TX) with a criterion of 3%/2 mm.

### C. Comparison of the calculated dose distributions

Patient dose distributions were calculated using the BrainsScan v.5.21 (BrainLAB) TPS. This TPS allows users to calculate the dose distributions of multiple beam datasets. Then, dose distributions were calculated for each patient by the TPS using the commissioning beam data provided by the X‐OMAT V2 radiographic and EBT2 radiochromic film. Both sets of the calculated dose distributions for each patient were exported for comparison. The metric used for the comparison of both calculated dose distributions for each patient was the gamma index criteria of 3%/3mm,2%/2mm,and1%/1mm using DoseLab. The difference in the half width (this difference is equal to the difference in the entrance distance to the brainstem) of the IDLs was also used to evaluate visual discrepancies between the dose distributions for each patient. In this work, the measured and calculated dose distributions are described according to the following regions: flat (100% to 80% IDLs), gradient (80% to 20% IDLs), and outer (<20%IDLs).

### D. Statistical analysis

**Figure 1:**
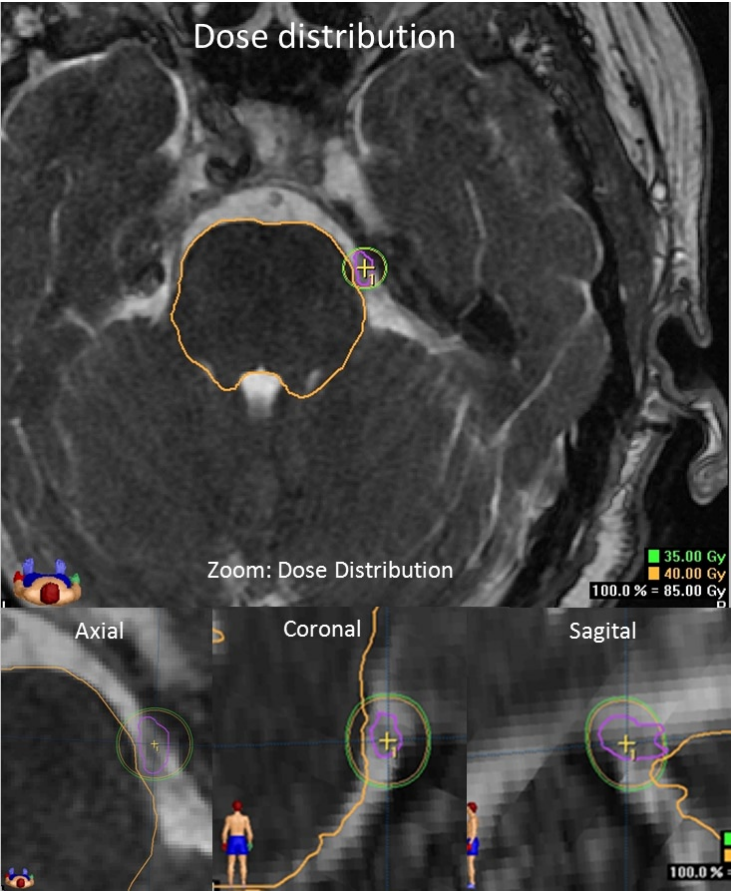
The dose distributions for TN. The IDLs of 35 and 40 Gy are shown. Notice the structures of interest involving a TN radiosurgery treatment: brainstem and trigeminal nerve. The contouring of the nerve is for isocenter placement only, since these treatments do not use the information provided by the dose volume histogram for its evaluation.

Certain parameters obtained from the TPS were chosen to compare how the calculated dose distributions may directly affect the observed dose delivered to the brainstem using two different sets of beam commissioning data: radiographic and radiochromic film. The first parameter was the volume of the brainstem that received a dose greater than or equal to 12 Gy (V12). The reported maximum brainstem dose of 12.5 Gy is associated with a low risk (<5%) of adverse effects.^(33)^ In addition, the entrance distances of two IDLs (35 and 40 Gy) into the brainstem were estimated by finding the shortest distance between the IDL and the brainstem contour (Fig. 1).

This procedure was performed for each patient using the commissioning data from both film detectors. Finally, the maximum dose received by the brainstem was calculated in the TPS for each patient using both commissioning datasets. The data were normally distributed according to the Kolmogorov‐Smirnov test. The differences in the parameters from each commissioning dataset were analyzed with paired t‐tests and considered to be significant when p<0.05.

## III. RESULTS

### A. Film measurements and comparison with stereotactic diode

**Figure 2:**
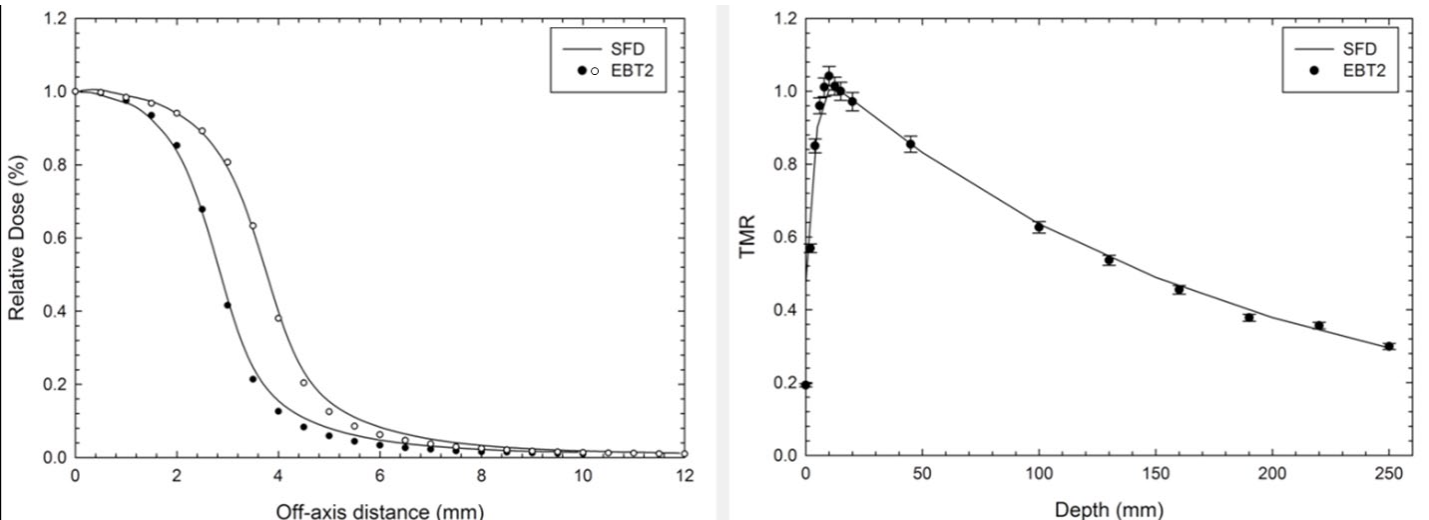
Relative measurements: the tissue maximum ratios and off‐axis ratios. For simplicity, the TMR for only 7.5 mm circular collimator is shown since TMR values are too close for the collimators used in this work. The presented values show a good agreement between the film and diode within statistical uncertainties.

A third‐order polynomial function was used to fit the film dose‐response curve, which resulted in a regression coefficient of 0.99. The TMR and OAR values were obtained and compared with the beam measurements made with a stereotactic diode (Fig. 2). The OFs measured with EBT2 film were 0.742 and 0.809 for the 6.0 and 7.5 circular collimators, respectively. The OFs measured with the stereotactic diode were 0.770 and 0.831 for the 6.0 and 7.5 circular collimators, respectively. The EBT2 beam measurements showed good agreement with the stereotactic diode measurements within the statistical uncertainties of both detectors. These uncertainties were 2.4% and 0.6% for the EBT2 and diode, respectively. These differences can be attributed to the diode response dependence on beam energy or to the film higher spatial resolution. However, establishing the nature of these differences is beyond the scope of this work, but it is necessary to make that clear in future investigations. Comparing the dose calculations of the TPS using the EBT2 commissioning dataset and EBT2 direct dose measurements in an end‐to‐end test, the gamma analysis showed that 100% of the evaluated pixels passed the 3%/2mm criteria. The mean gamma value was 0.1, indicating good agreement between the calculated and measured dose distributions.

### B. Dose distributions comparison

**Figure 3:**
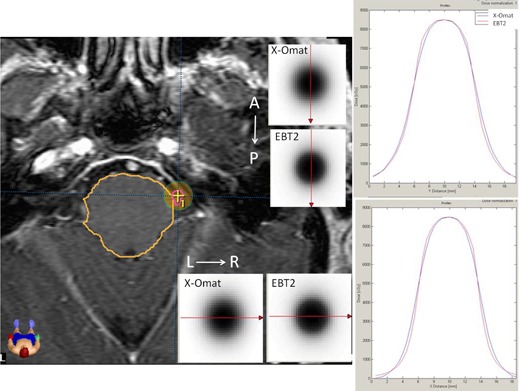
The horizontal and vertical dose profiles of the calculated dose distributions by the TPS. The subfigures are axial planes located at isocenter that shows TPS‐calculated dose distributions for both sets of dosimetry data (EBT2 and X‐Omat). The arrows show the direction of the profiles: left—right (LR) and anterior‐posterior (AP).

The results of the gamma index comparison showed that all of the patients (23; 100%) passed the 3%/3mm criteria, 22 (95.6%) passed the 2%/2mm criteria, and only seven (30.4%) passed the 1%/1mm criteria. In Fig. 3, the horizontal and vertical dose profiles show the systematic differences between both of the calculated dose distributions. In the flat region and part of the gradient region, the calculated dose distribution based on the radiographic commissioning beam data showed a “shorter” profile than the profile based on the EBT2 beam data. In contrast, in the outer region and part of the gradient region, the profile observation showed the opposite behavior. An analysis of the same profiles for all of the patients showed that the half widths of the profiles for the 35 and 40 Gy IDLs exhibited differences less than or equal to 0.58 and 0.74 mm, respectively. For these IDLs, the radiographic‐based profiles were wider than the EBT2‐based profiles (see Fig. 3).

### C. Statistical analysis

When comparing the maximum entrance distances estimated for the 35 and 40 Gy isodose curves in the brainstem from both commissioning datasets, it was found that the entrance distances were smaller when using the radiochromic film dataset. The differences in the maximum entrance distances between both datasets for the 35 and 40 Gy IDLs were 0.33 mm (p<0.0001) and 0.38mm(p<0.0001), respectively. When using the EBT2 commissioning dataset, the V12 was 6.25% (2.1 cc) lower (p=0.022) than when using the radiographic commissioning dataset. The maximum dose received by the brainstem was not statistically significantly different for the two dataset distributions.

## IV. DISCUSSION & CONCLUSION

For the same set of TN patients, the dose distributions calculated with a commissioning dataset provided by X‐OMAT V2 radiographic film were compared with the dose distributions calculated with the dosimetry of EBT2 radiochromic film using the same TPS. The results showed systematic differences between the calculated dose distributions based on the commissioning beam data for each film detector. The observed differences can be attributed to the radiographic film's effective atomic number (mainly AgBr) compared to the near tissue equivalence of the radiochromic film.^(16)^ Because of the multiple interactions from charged particles generated in the films after irradiation, the energy loss tends to be different due to the effective atomic number of both films. The stopping power decreases as the effective atomic number increases. Thus, the energy and dose deposition on the film vary according to the components of the film, causing the difference in dose distributions. Accordingly, EBT2 radiochromic film has a lower energy dependence than radiographic film. Although a study such as this cannot prove that the commissioning dataset from EBT2 film is closer to the true dose distribution, there are statistically significant differences in the usage of X‐OMAT V2 radiographic film and EBT2 radiochromic film, particularly for the 35 and 40 Gy IDL entrance distances into the brainstem and the brainstem volumes that receive more than 12 Gy. The magnitudes of these differences were found to be less than or equal to the voxel size of the CT volume used for the treatment planning. In clinical practice, it seems unlikely that these differences could affect the evaluation of a TN treatment plan based on these IDLs. However, this work showed that there was a significant difference (mean of 2.1 cc) between both of the calculated dose distributions regarding the V12. It is clear that these differences may impact the treatment of TN patients, and then it must be important to perform this type of analysis when observing complication rates. Clinical reports on irradiation techniques for trigeminal neuralgia should consider that different detectors used for commissioning treatment planning systems might result in small but significant differences in dose distributions.

## References

[1] Jursinic P , Rickert K , Gennarelli T , Schultz C . Effect of image uncertainty on the dosimetry of trigeminal neuralgia irradiation. Int J Radiat Oncol Biol Phys. 2005;62(5):1559–67. 16029818 10.1016/j.ijrobp.2005.01.059

[2] Goss B , Frighetto L , De Salles A , Smith Z , Solberg T , Selch M . Linear accelerator radiosurgery using 90 gray for essential trigeminal neuralgia: results and dose volume histogram analysis. Neurosurgery. 2003;53(4):823–30. 14519214 10.1227/01.neu.0000083550.03928.d8

[3] Richards G , Bradley K , Tom&eacute; W , Bentzen S , Resnick D , Mehta M . Linear accelerator radiosurgery for trigeminal neuralgia. Neurosurgery. 2005;57(6):1993–200. 10.1227/01.neu.0000186015.01179.70 16331167

[4] Maesawa S , Salame C , Flickinger JC , Piris S , Kondziolka D , Lundsford LD . Clinical outcomes after stereotactic radiosurgery for idiopathic trigeminal neuralgia. J Neurosurg. 2001;94(1):14–20. 11147887 10.3171/jns.2001.94.1.0014

[5] Pollock BE , Phuong LK , Gorman DA , Foote RL , Stafford SL . Stereotactic radiosurgery for idiopathic trigeminal neuralgia. J Neurosurg. 2002;97(2):347–53. 12186463 10.3171/jns.2002.97.2.0347

[6] Rand RW . Leksell gamma knife treatment of tic doulourex. Neurosurg Clin N Am. 1997;8(1):75–78. 9018707

[7] Rand RW , Jacques DB , Melbye RW , Copcutt BG , Levenick MN , Fisher MR . Leksell gamma knife treatment of tic doulourex. Stereotact Funct Neurosurg. 1993;61(Supp 1):93–102. 8115760 10.1159/000100663

[8] Regis J , Bartolomei F , Metellus P , et al. Radiosurgery for trigeminal neuralgia and epilepsy. Neurosurg Clin N Am. 1999;10(2):359–76. 10099103

[9] Rogers CL , Shelter AG , Fiedler JA , Smith KA , Han PP , Speiser BL . Gamma Knife radiosurgery for trigeminal neuralgia: the initial experience of the Barrow Neurological Institute. Int J Radiat Oncol Biol Phys. 2000;47(4):1013–19. 10863073 10.1016/s0360-3016(00)00513-7

[10] Young RF , Vermeulen SS , Grimm P , Blasko J , Posewitz A . Gamma Knife radiosurgery for treatment of trigeminal neuralgia. Neurology. 1997;48(3):608–14. 9065534 10.1212/wnl.48.3.608

[11] Ma L , Kwok Y , Chin L , Yu W , Regine W . Comparative analyses of linac and Gamma Knife radiosurgery for trigeminal neuralgia treatments. Phys Med Biol. 2005;50(22):5217–27. 16264249 10.1088/0031-9155/50/22/001

[12] Paskalev K , Seuntiens J , Patrocinio H , Podgorsak E . Physical aspects of dynamic stereotactic radiosurgery with very small photon beams (1.5 and 3 mm in diameter). Med Phys. 2003;30(2):111–18. 12607827 10.1118/1.1536290

[13] Wilcox E and Daskalov G . Accuracy of dose measurements and calculations within and beyond heterogeneous tissues for 6 MV photon fields smaller than 4 cm produced by Cyberknife. Med Phys. 2008;35(6):2259–66. 18649456 10.1118/1.2912179

[14] Wu A , Zwicker RD , Kalend AM , Zheng Z . Comments on dose measurements for a narrow beam in radiosurgery. Med Phys. 1993;20(3):777–79. 8350836 10.1118/1.597032

[15] Hardcastle N , Basavatia A , Bayliss A , Tom&eacute; W . High dose per fraction dosimetry of small fields with Gafchromic EBT2 film. Med Phys. 2011;38(7):4081–85. 21859007 10.1118/1.3597834

[16] GAFCHROMIC$rg EBT2: Self‐developing film for radiotherapy dosimetry. Wayne, NJ: International Speciality Products; 2009.

[17] Inhwan Jason Yeo , Jong Oh Kim . A procedural guide to film dosimetry with emphasis on IMRT. Madison, WI: Medical Physics Publishing; 2004 p. 2–1–2–7.

[18] Schell MC , Bova FJ , Larson DA , et al. AAPM report No.54. Stereotactic radiosurgery. Report of Task Group 42 Radiation Therapy Committee. College Park, MD: American Institute of Physics; 1995.

[19] Gorgulho AA and De Salles AAF . Impact of radiosurgery on the surgical treatment of trigeminal neuralgia. Surg Neurol. 2006;66(4):350–56. 17015103 10.1016/j.surneu.2006.03.046

[20] BrainScan Manual. Software Guide, Revision 1.0. Feldkirchen, Germany: BrainLab AG; 2004.

[21] Pai S , Das IJ , Dempsey JF , et al. Task Group 69: radiographic film for megavoltage beam dosimetry. Med Phys. 2007;34(6):2228–58. 17654924 10.1118/1.2736779

[22] Niroomand‐Rad A , Blackwell C , Coursey B , et al. AAPM Report No. 63. Radiochromic film dosimetry. Recommendations of AAPM Radiation Therapy Committee Task Group 55. Med Phys. 1998;25(11):2093–115. 9829234 10.1118/1.598407

[23] Butson MJ , Cheung T , Yu PKN . Scanning orientation effects on Gafchromic EBT film dosimetry. Australas Phys Eng Sci Med. 2006;29(3):281–84. 17058592 10.1007/BF03178579

[24] Lynch BD , Kozelka J , Ranade MK , Li JG , Simon WE , Dempsey JF . Important considerations for radiochromic film dosimetry with flatbed CCD scanners and EBT GAFCHROMIC film. Med Phys. 2006;33(12):4551–56. 17278806 10.1118/1.2370505

[25] Richley L , John AC , Coomber H , Fletcher S . Evaluation and optimization of the new EBT2 radiochromic film dosimetry system for patient dose verification in radiotherapy. Phys Med Biol. 2010;55(9):2601–17. 20393235 10.1088/0031-9155/55/9/012

[26] Aldelaijan S , Devic S , Mohammed H , et al. Evaluation of EBT‐2 model GAFCHROMIC film performance in water. Med Phys. 2010;37(7):3687–93. 20831076 10.1118/1.3455713

[27] Rasband WS . ImageJ. Bethesa, MD: US National Institute of Health; 1997.

[28] Devic S , Seuntjens J , Sham E , et al. Precise radiochromic film dosimetry using a flat‐bed document scanner. Med Phys. 2005;32(7):2245–53. 10.1118/1.1929253 16121579

[29] Garcáa‐Garduño OA , Celis Lopez MA , Lárraga‐Gutiérrez JM , Moreno‐Jiménez S , Martánez‐Dávalos A , Rodráguez‐Villafuerte M . Radiation transmission, leakage and beam penumbra measurements of a micro‐multileaf collimator using GafChormic EBT film. Int J Appl Clin Med Phys. 2008;9(3):90–98. 10.1120/jacmp.v9i3.2802 PMC5722293 18716595

[30] International Atomic Energy Agency. Absorbed dose determination in external beam radiotherapy. Technical Reports Series No. 398. Vienna: International Atomic Energy Agency; 2000.

[31] Galván De la Cruz OO , Garcáa Garduño OA , Hernández Reyez B , Jimenez SM , Celis Lopez MA , Larraga‐Gutierrez JM . Measurement of entrance skin dose due to the imaging system for treatment planning of stereotactic radiosurgery of arteriovenois malformations. WC 2009, IFMBE Proceedings, Vol. 25 New York: Springer Verlag; 2009 p. 390–92.

[32] Low D , Harms W , Mutic S , Purdy J . A technique for the quantitative evaluation of dose distributions. Med Phys. 1998;25(5):656–61. 9608475 10.1118/1.598248

[33] Mayo C , Yorke E , Mechant T . Radiation associated brainstem injury. Int J Radiat Oncol Biol Phys. 2010;76(3 Suppl):S36–S41. 20171516 10.1016/j.ijrobp.2009.08.078 PMC2899702

